# Seamless monotherapy-combination phase I dose-escalation model-based design

**DOI:** 10.1177/17407745251350604

**Published:** 2025-07-12

**Authors:** Libby Daniells, Thomas Jaki, Alimu Dayimu, Nikos Demiris, Basu Bristi, Stefan Symeonides, Pavel Mozgunov

**Affiliations:** 1 MRC Biostatistics Unit, University of Cambridge, Cambridge, UK; 2 Faculty of Informatics and Data Science, University of Regensburg, Regensburg, Germany; 3 Clinical Trials Unit, Cancer Theme, University of Cambridge, Cambridge, UK; 4 Department of Oncology, University of Cambridge, Cambridge, UK; 5 Edinburgh Experimental Cancer Medicine Centre, Institute of Genetics and Cancer, The University of Edinburgh, Edinburgh, UK; 6 Centre for Drug Development, Cancer Research UK, London, UK

**Keywords:** Dose-finding, combination study

## Abstract

Phase I dose-escalation studies for a single-agent and combination of anti-cancer agents have explored various model-based designs to guide identification of a maximum tolerated dose and recommended phase II dose. This work describes a parallel approach to dose escalation to expedite identification of maximum tolerated doses both for an anti-cancer agent as monotherapy and in combination with another agent. We develop a three-parameter Bayesian logistic regression model that allows for more efficient use of information between monotherapy and combination parts of the study. The model allows the monotherapy and combination data to drive dose escalation of the combination of treatments, reflecting the known dose-toxicity relationship between the monotherapy and combination setting. Through a thorough simulation study in which the proposed model is compared to two comparative approaches, the three-parameter Bayesian logistic regression model is shown to accurately select doses in the target toxicity interval, performing similar to comparative approaches in terms of proportion of target dose/combination selection, while more than halving the proportion of doses selected that were greater than the target toxicity, thereby improving safety concerns.

## Introduction

In a phase I dose-escalation study, the traditional primary objective is to determine a maximum tolerated dose (MTD). The MTD is defined as the highest dose that harbours an acceptable level of toxicity.^1^ This toxicity is determined by the probability of an unacceptable adverse event related to the treatment. These events are referred to as dose-limiting toxicities (DLTs). Dose-finding studies commonly rely on a monotonicity assumption, meaning that a higher dose level will result in a greater clinical benefit compared to a lower dose. However, it is also assumed that a higher dose will result in a greater toxicity risk. Thus, the goal of the early-phase study is to explore the dose-toxicity relationship by escalating/de-escalating through the doses, ultimately identifying the MTD.

These trials have predominantly been implemented in the monotherapy setting in which the study investigates a single therapeutic treatment with interest lying in identifying the MTD of the monotherapy.^2^ However, increasingly common in the immunotherapy setting in particular, is the investigation of an active agent in combination with a backbone agent. In this setting, dose escalation only concerns the dose-level of the active agent under investigation, while the dose of the backbone agent is fixed. If the backbone agent were to have multiple dose levels under investigation, there are a plethora of existing methods in the literature.^3–9^ The model proposed in this work has been developed for a single dose level for the backbone agent as motivated by Cancer Research UK’s current Phase I/IIa trial of HTL0039732.^10^ HTL0039732 (or NXE0039732), from Nxera Pharma, is a small molecule antagonist of prostaglandin PGE2’s receptor EP4 that has been designed to block EP4-driven immunosuppressive effects within the tumour microenvironment and to enhance the activity of existing immunotherapies such as programmed cell death protein 1 (PD-1)/programmed death ligand 1 (PD-L1) checkpoint inhibitors, including atezolizumab. This motivates the development of a unique trial design that can efficiently conduct both dose escalation under monotherapy *and* dose escalation of the treatment in combination with the backbone agent.

In this article, we propose a novel dose-escalation model that enables the parallel testing of a treatment as a monotherapy alongside the dose escalation of the treatment in combination with a fixed dose of a backbone agent. The goal of the study is to find the MTD at monotherapy (where the MTD is defined as the dose that maximises the probability of the toxicity risk being in the target toxicity interval), as well as the maximum tolerated combination (MTC) of the treatment with the backbone agent. This relies on the assumption of monotonicity of toxicity under monotherapy, with a higher dose assumed to be more toxic than a lower dose, but also assumes that a treatment administered in combination with a backbone agent is as/more toxic than the same dose administered as monotherapy. By conducting parallel testing of the monotherapy and combination as opposed to sequential testing, the study efficiency is greatly improved. Not only will this reduce the duration of the study, but it will also allow for more efficient use of information between the monotherapy and combination parts of the study. The proposed model builds on the two-parameter Bayesian logistic regression model (BLRM) proposed by Neuenschwander et al.^11^ by incorporating a third parameter to model the combination with the backbone agent. We compare the performance of the model to two alternative models: the partial-ordering continual reassessment method (POCRM)^12^ and the two-parameter BLRM^11^ that conducts the dose escalation of monotherapy and in combination sequentially. The proposed three-parameter model is demonstrated to balance both accuracy of selection and safety concerns, with a substantial improvement in the selection of over-toxic doses compared to the two comparative models.

Despite the plethora of efficient statistical models for phase I dose-finding studies, there is a severe lack in their uptake for implementation in practice.^8^ Through the work presented in this article, we hope to build on the growing literature for the implementation of more complex dose escalation models and to demonstrate the practical and statistical advantages it holds.

## Methodology

### Notation

In this section, notation is defined and the novel three-parameter BLRM for dose escalation outlined. Let there be a total of J discrete doses under investigation, with doses denoted $d_{j}$ for j=1,…,J . The risk of a DLT under dose $d_{j}$ is defined as $p\left(d_{j},f\right)$ , where f is a categorical variable denoting whether the treatment was administered as a monotherapy or in combination with the backbone agent. Due to the monotonicity assumption, $p\left(d_{j},\mathrm{monotherapy}\right)$ increases as the dose, $d_{j}$ , of monotherapy increases and $p\left(d_{j},\mathrm{combination}\right)$ increases as the dose, $d_{j}$ , under the combination therapy increases. In addition, $p\left(d_{j},\mathrm{combination}\right)\geq p\left(d_{j},\mathrm{monotherapy}\right)\;\forall i=1,\ldots ,J$ , that is, a dose at combination is always never less toxic than the same dose at monotherapy. The maximum number of patients on the trial across both monotherapy and combination is denoted by N , with a maximum of $n_{\mathrm{mono}}$ on the monotherapy and $n_{\mathrm{comb}}$ on the combination treatment.

### Proposed three-parameter Bayesian logistic regression dose-escalation model

Neuenschwander et al.^11^ proposed a two-parameter BLRM which is adapted to incorporate the escalation of the dose of a single agent (monotherapy) alongside the dose escalation of this single agent administered in combination with a backbone agent (combination), into a single model. The adaptation incorporates an additional binary covariate to the two-parameter BLRM. This is a similar model to that of Bailey et al.;^13^ however, their approach was not implemented to escalate both monotherapy and combination therapy in parallel.

The proposed model is a three-parameter BLRM where the extra parameter indicates whether the agent is administered as a monotherapy or in combination. The model has the following form

(1) $$\mathrm{logit}\left(p\left(d_{j},f\right)\right)=\alpha_{0}+\alpha_{1}\times \log\left(d_{j}/D_{*}\right)+\alpha_{2}\times I[f],$$

where $D_{*}$ is a reference dose. The indicator function I[f]=I[monotherapy]=0 when f denotes that the monotherapy treatment is administered and I[f]=I[combination]=1 when f denotes that a combination therapy is administered. The parameters $\alpha_{0}$ and $\alpha_{1}$ are the intercept and slope parameters of the model. A normal prior distribution is placed on these parameters: $\left(\alpha_{0},\log\left(\alpha_{1}\right)\right)\;\sim \;N\left(\mu ,\Sigma \right)$ , where $\mu ={\left(\mu_{0},\mu_{1}\right)}^{T}$ is the vector of means and

$$\Sigma =\left[\begin{array}{c} \sigma_{0}^{2}\sigma_{01}\\ \sigma_{01}\sigma_{1}^{2} \end{array}\right]$$

is the covariance matrix. The reference dose $D_{*}$ is implemented for the sake of interpretability of these model parameters, with the $\alpha_{0}$ term being the odds of a DLT at $D_{*}$ . The $\alpha_{2}$ parameter, corresponding to the combination therapy, also follows a normal distribution: $\;\log\left(\alpha_{2}\right)\;\sim \;N\left(\mu_{2},\sigma_{2}^{2}\right)$ . The covariate term is restricted to be strictly positive to reflect the assumption that the combination cannot decrease the risk to toxicity when compared to administering the same dose at monotherapy.

The monotherapy and combination therapy dose escalation can run in parallel under this model. The addition of the indicator function means that only the $\alpha_{0}$ and $\alpha_{1}$ parameters drive the dose escalation for monotherapy. However, in the combination part of the study, I[combination]=1 and thus all three model parameters $\alpha_{0},\;\alpha_{1}$ and $\alpha_{2}$ contribute to dose escalation for the combination part. In this way, the monotherapy data does not contribute to the estimate of the combination term, $\alpha_{2}$ , with the prior distribution $\left(\alpha_{0},\log\left(\alpha_{1}\right)\right)$ updated by the monotherapy data only. However, the combination data do contribute to all model parameters, with the prior distribution of $\left(\alpha_{0},\log\left(\alpha_{1}\right),\left(\alpha_{2}\right)\right)$ updated with the combination data.

**Figure 1.**
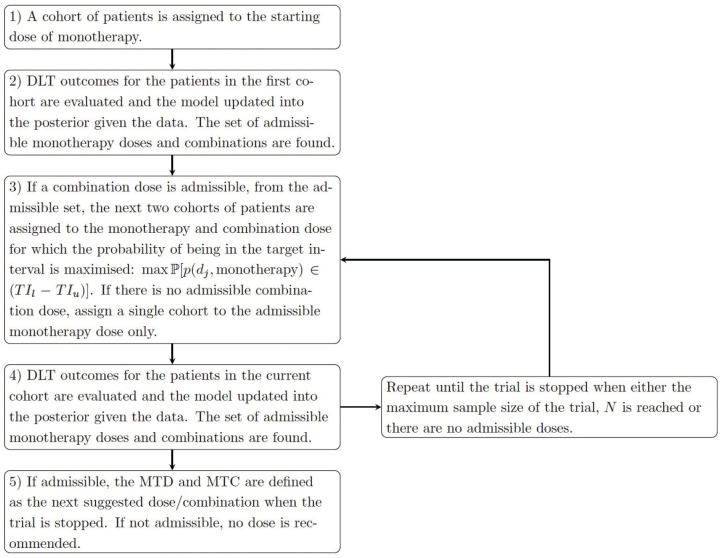
Dose escalation under the proposed three-parameter BLRM.

Figure 1 demonstrates how a dose-escalation trial is conducted under this proposed three-parameter BLRM, for the parallel testing of monotherapy and combination therapy. The trial commences with a cohort of patients assigned to the starting dose of monotherapy. Their DLT outcomes are observed and are used to update the model, which is then used for dose escalation recommendation. From the model, the next cohort of patients will be assigned to the highest admissible dose that maximises the probability of the risk of a DLT being in the target toxicity interval of $TI_{l}-TI_{u}$ . To find the admissible doses at each stage we implement several dose-escalation rules:

- Doses cannot be skipped;
- A dose of the treatment given in combination can only be assigned from the second cohort onwards (given that the dose-escalation rules are satisfied), allowing the accrual of some data for the monotherapy before administering in combination.
- A dose of the treatment given in combination can only be assigned if there have been at least three DLT-evaluated patients on this (or higher) dose as a monotherapy;
- If one out of three patients in the current cohort had a DLT event, then the next cohort of patients cannot receive a dose that is higher than the current dose;
- Patients can only be assigned to doses/combinations that are deemed safe. A dose/combination is deemed safe if the probability of the risk of toxicity exceeding the upper toxicity threshold of $TI_{u}\%\;$ is below a fixed overdosing constant, $c_{\mathrm{overdose}}$ . Formally

$$P[p\left(d_{j},f\right)\geq TI_{u}\%]\leq c_{\mathrm{overdose}},$$

where $p\left(d_{j},\;f\right)$ is the probability of a DLT at dose $d_{j}$ conditional on f , where f= combination denotes that the monotherapy was administered alongside the backbone agent and f= monotherapy when administered on its own.

Any dose or combination which satisfied all four of the constraints are referred to as an admissible dose/combination. Dose escalation/de-escalation can only occur between admissible doses. The trial terminates when either no doses are deemed admissible (for safety concerns) or if the total maximum sample sizes of $n_{\mathrm{mono}}$ and $n_{\mathrm{comb}}$ for monotherapy and combination therapy have been reached.

Throughout the rest of this work, the proposed three-parameter BLRM is compared to two comparative methods: the two-parameter BLRM (which is equivalent to the proposed design when I[f]=0 ) and the POCRM. The details of these models and how they are implemented in this setting are provided in the supplementary materials.

## Example trial

In the example trial, an experimental treatment is administered to patients with advanced cancers. The study includes a small number of patients in order to determine the safety and tolerability of the treatment. In total, it is assumed that seven doses will be explored: 20 mg, 40 mg, 80 mg, 160 mg, 320 mg, 640 mg and 960 mg, where 20 mg and 40 mg are de-escalation doses only and 80 mg is the starting dose. Each dose is administered orally on a daily 21-day schedule with no breaks and the DLTs are assessed over several cycles. The trial is split into two parts: Part A in which doses of the treatment are administered as a monotherapy and Part B in which doses of the treatment are administered alongside intravenous atezolizumab as a backbone agent. The maximum number of patients to be enrolled in the study (in the monotherapy and combination parts) is assumed N=36 but could be flexible depending on the accumulated data. The MTD and MTC in this setting are defined as the highest safe dose/combination which maximises the probability of the risk of a DLT being in the target toxicity interval of 20%–30%.

The goal is to implement a dose-escalation model that can efficiently explore the dose space and to ultimately determine both the MTD and the MTC as quickly as possible. As such, the proposed design is to conduct the monotherapy dose escalation and the dose escalation of the treatment in combination with atezolizumab in parallel. This will reduce the duration of the trial and more efficiently utilise the accrued monotherapy and combination data.

### Example model outputs for the proposed design

**Figure 2.**
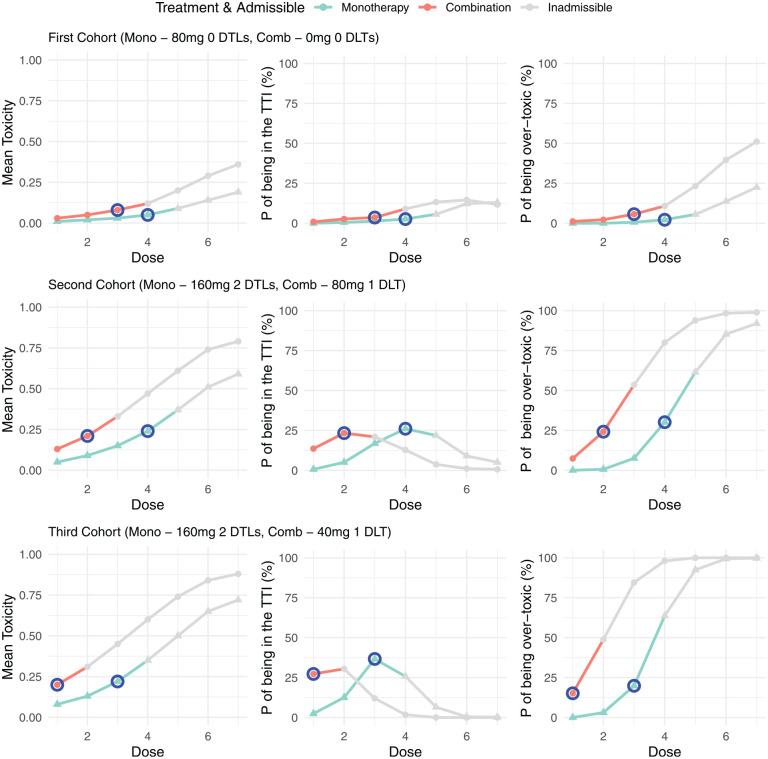
Example trial 1: model outputs for the first three cohorts of a trial. For each cohort the mean toxicity, probability of being in the target toxicity interval (TTI) and the probability of being over-toxic is presented for each dose under both monotherapy and combination treatment. Doses that are inadmissible are greyed out, with the next allocated dose for both monotherapy and combination highlighted in blue.

Given the above trial setting, we now demonstrate the model outputs for the first three cohorts under two example data sets. For each cohort, the mean toxicity, probability of being in the target toxicity interval and the probability of being over-toxic is presented for every dose under both monotherapy and combination treatment. The first example is presented in Figure 2, where the first cohort were only administered the monotherapy, with the combination treatment not yet started. In this cohort no DLTs were observed. This information was used to update the model and obtain the toxicity risk for doses at both monotherapy and combination. The model recommends increasing the monotherapy dose to 160 mg, with all higher doses inadmissible due to the escalation rule that no doses may be skipped. The model also indicates that the first three doses at combination are admissible and selects 80 mg as the dose for the first combination cohort. This process is repeated in cohorts 2 and 3, with the doses for both monotherapy and combination remaining the same or de-escalated based on the DLTs observed.

**Figure 3.**
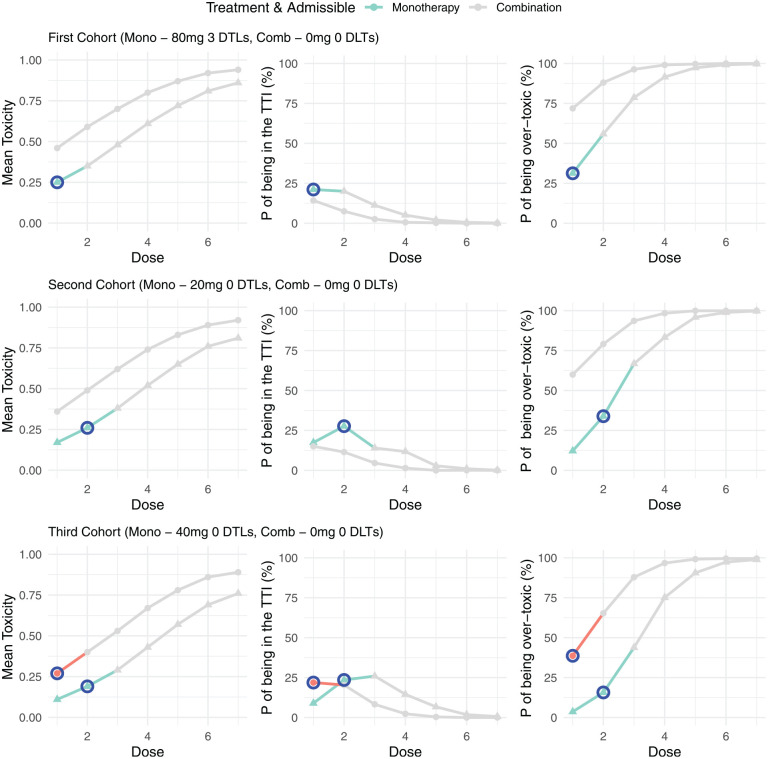
Example trial 2: model outputs for the first three cohorts of a trial. For each cohort the mean toxicity, probability of being in the target toxicity interval (TTI) and the probability of being over-toxic is presented for each dose under both monotherapy and combination treatment. Doses that are inadmissible are greyed out, with the next allocated dose for both monotherapy and combination highlighted in blue.

In the second data set, presented in Figure 3, three DLTs were observed in the first cohort so only the first monotherapy dose was admissible. As a result, the second cohort was assigned to a dose of 20 mg. Unlike in example 1, combination treatment did not start in cohort 2, as no combination doses are admissible. In the second cohort, no DLTs were observed and the monotherapy dose was escalated to 40 mg, while still no combinations were deemed admissible. It is only after the third cohort that a combination dose was an admissible dose and so dose escalation for the combination will start in parallel from cohort 4 onwards.

## Calibration of model parameters

The proposed model has a number of prior parameters. Before conducting the trial, each of these parameters are calibrated in order to produce favourable operating characteristics across several quantitatively and qualitatively different dose-toxicity scenarios. This is conducted via extensive simulation studies in order to optimise the accuracy of dose escalation and MTD/MTC selection.

### Setting

The simulation setting considered here is one in which both monotherapy and combination therapy is explored in parallel. Results of simulations under monotherapy alone are provided in the supplementary materials.

From the motivating example, we now define a dose-escalation trial setting used throughout the rest of this article. For all three approaches, the starting dose was set at 80 mg. At each stage of dose escalation, a cohort consisting of three patients were enrolled onto a dose. The trial is terminated when either the maximum total number of N=36 patients (a maximum of $n_{\mathrm{mono}}=24$ on the monotherapy and $n_{\mathrm{comb}}=12$ on the combination treatment) were assigned to a dose or if the safety constraint was violated for all doses. The safety constraint is defined as: $P[p\left(d_{j},f\right)\geq 30\%]\leq c_{\mathrm{overdose}}$ , where $p\left(d_{j},f\right)$ is the probability of the risk of toxicity at dose $d_{j}$ dependent on whether a monotherapy or combination treatment is administered. Thus, a dose is deemed unsafe if this safety constraint is violated. If the trial has not been terminated early based on the safety constraint, then the MTD is defined as the dose that maximises the probability of the toxicity risk being in the target toxicity interval, that is, $\;\max\;P[\;p\left(d_{j},f\right)\in \left(20-30\%\right)]$ at the end of the trial. For the BLRM, a reference dose of $D_{*}$ = 960 mg was implemented based on statistical considerations. A 2:1 allocation ratio between monotherapy and combination treatment was implemented as the monotherapy was a new experimental treatment, and thus, the focus was not only on dose escalation and safety but also on other properties such as pharmacokinetic profile, which required a larger sample size.

### Calibration of monotherapy parameters

First consider the setting where patients are assigned to the monotherapy treatment, with no combination dose escalation considered; thus, the maximum sample size is $N=n_{\mathrm{mono}}=24$ patients enrolled across the doses. In this monotherapy setting, seven dose-toxicity scenarios were implemented for calibration across. These scenarios are presented in Table 1, where values represent the true toxicities for each dose. These scenarios are defined such that the upper limit of the target toxicity interval, 30%, is $d_{1}$ in scenario 1, $d_{2}$ in scenario 2 and so on.

**Table 1.** Considered dose-toxicity scenarios for monotherapy treatment.

Scenario	$d_{1}$	$d_{2}$	$d_{3}$	$d_{4}$	$d_{5}$	$d_{6}$	$d_{7}$
Scenario 1	0.30	0.45	0.55	0.60	0.65	0.70	0.75
Scenario 2	0.20	0.30	0.45	0.55	0.60	0.65	0.70
Scenario 3	0.15	0.20	0.30	0.45	0.55	0.60	0.65
Scenario 4	0.10	0.15	0.20	0.30	0.45	0.55	0.60
Scenario 5	0.05	0.10	0.15	0.20	0.30	0.45	0.55
Scenario 6	0.02	0.05	0.10	0.15	0.20	0.30	0.45
Scenario 7	0.00	0.02	0.05	0.10	0.15	0.20	0.30

Doses within the target toxicity interval of 20%–30% are highlighted in **bold**.

The proposed three-parameter model consists of several parameters: $\left(\mu_{0},\mu_{1},\mu_{2},\sigma_{0},\sigma_{1},\sigma_{2},c_{\mathrm{overdose}}\right)$ . However, in the monotherapy setting, this can be reduced down to $\left(\mu_{0},\mu_{1},\sigma_{0},\sigma_{1},c_{\mathrm{overdose}}\right)$ as the combination term is set to 0. The calibration occurs using a grid-search approach, where the candidate parameter values for $\mu_{0},\;\mu_{1},\;\mu_{2},\;\sigma_{0},\;\sigma_{1}\;\mathrm{and}\;\sigma_{2}$ are selected to construct substantially different dose-toxicity relationships.^14^ For the overdose parameter, $c_{\mathrm{overdose}}$ , Barnett et al.^9^ demonstrate that as $c_{\mathrm{overdose}}$ changes, there is a trade-off of between the increase in over-toxic dose selection and the number of correct dose selected. Ewings et al.^14^ state that a value of $c_{\mathrm{overdose}}=0.25$ has demonstrated good safety properties when the two-parameter BLRM is applied. Thus, the candidate values are selected as 0.25 or higher, as higher values will explore the trade-off in increased over-toxic selections and probability of correct selections. The following parameters were implemented for the grid-search in our setting:

1. Hyper-parameters for the intercept $\mu_{0}=\{-2.75,-2.50,\ldots ,-1.50\}$ , $\sigma_{0}=\{1.20,1.40,\ldots ,2.20\}$ ;
2. Hyper-parameters for the slope, $\mu_{1}=\{-0.50,-0.25,\ldots ,0.75\}$ , $\sigma_{1}=\{0.20,0.35,\ldots ,0.80\}$ ;
3. Overdosing probability threshold $c_{\mathrm{overdose}}=\{0.25,0.30,0.35,0.40,0.45\}$ ,

with the covariance parameter set to 0. For each combination of these parameter values, the probability of selecting the dose with the risk of toxicity within the target interval (selection of the doses with a DLT risk between 20% and 30%) is computed under each of the seven dose-toxicity scenarios using 500 simulation runs for each combination and scenario. Simulations were conducted using the ‘rjags’ package^15^ in R Studio^16^ version 4.1.1. Only parameter combinations that satisfy the following dose-escalation constraints are considered in the calibration:

- If 0/3 DLTs are observed in the first cohort, 160 mg should be safe;
- If 1/3 DLTs are observed in the first cohort, 80 mg should be safe;
- If 2/3 DLTs are observed in the first cohort, the dose must be de-escalated and 40 mg should be safe.
- If 3/3 DLTs are observed in the first cohort, the dose must be de-escalated and 40 mg should be unsafe and 20 mg should be safe.
- The combination of parameter values that imply good operating characteristics across all scenarios, while satisfying the above constraints will be taken forward for further evaluation. This is quantified by taking the geometric mean of the probability of correct selection of an MTD in the target toxicity interval across each of the seven dose-toxicity scenarios. Through this calibration, the combination of parameter values selected were: $\mu_{0}=-1.75,\sigma_{0}=1.6,\;\mu_{1}=0,\;\sigma_{1}=0.2$ and $c_{\mathrm{overdose}}=0.4$ .

The dose-escalation behaviour of the calibrated monotherapy model can be evaluated through decision trees that show the recommended doses for different cohorts of patients dependent on the observed number of DLTs. For details on this please refer to the supplementary materials.

### Calibration of combination therapy parameters

In order to calibrate the parameter values for the combination parameter in the proposed three-parameter BLRM, the calibrated values for $\left(\mu_{0},\mu_{1},\sigma_{0},\sigma_{1},c_{\mathrm{overdose}}\right)$ as obtained under the monotherapy setting alone are implemented. This leaves the calibration of the $\left(\mu_{2},\sigma_{2}\right)$ parameters which correspond to the experimental treatment given in combination with the backbone agent. As in the monotherapy setting, the parameter space is searched and calibrated values selected based on operating characteristics and performance.

To conduct the calibration, combination-toxicity scenarios must be constructed. These scenarios are used as the true probabilities of a DLT under the combination treatment at each dose. For the monotherapy treatment, the scenarios in Table 1 are implemented for the true probabilities of a DLT. The monotonicity assumption is used to construct combination-toxicity scenarios from these monotherapy scenarios, wherein the dose at combination can be no less toxic than the same dose at monotherapy. As such, for each monotherapy scenario in Table 1, several possible combination-toxicity scenarios are considered, where the true MTC lies between 1 and 3 doses lower than the MTD at monotherapy. Formally, under monotherapy scenario x ( x=1,2,…,7 ), the combination-toxicity scenario with the MTC being y ( y=1,2,3 ) doses below the MTD under monotherapy is denoted as scenario x.y . Two example scenarios are presented in Table 2, the first of which is monotherapy scenario 4, where the target toxicity interval is between $d_{3}$ and $d_{4}$ . When considering the combination setting, the dose-toxicity values under scenario 4 are applied as the true probabilities of a DLT for the monotherapy treatment, while three different scenarios are considered for the probabilities of a DLT under combination treatment. The first of which is scenario 4.1, where the target toxicity interval is one dose below the monotherapy target toxicity interval, lying between $d_{2}$ and $d_{3}$ , second scenario 4.2 where the target toxicity interval is two doses below, lying between $d_{1}$ and $d_{2}$ , and so on. The combination-toxicity scenarios for monotherapy scenarios 1–7 are constructed in the same way. All combination-toxicity scenarios can be found in the supplementary materials.

**Table 2.** Considered dose-toxicity and combination-toxicity scenarios.

Scenario	$d_{1}$	$d_{2}$	$d_{3}$	$d_{4}$	$d_{5}$	$d_{6}$	$d_{7}$
Scenario 4 (Monotherapy)	0.10	0.15	0.20	0.30	0.45	0.55	0.60
Scenario 4.1 (Combination)	0.15	0.20	0.30	0.45	0.55	0.60	0.65
Scenario 4.2 (Combination)	0.20	0.30	0.45	0.55	0.60	0.65	0.70
Scenario 4.3 (Combination)	0.30	0.45	0.55	0.60	0.65	0.70	0.75
Scenario 7 (Monotherapy)	0.00	0.02	0.05	0.10	0.15	0.20	0.30
Scenario 7.1 (Combination)	0.02	0.05	0.10	0.15	0.20	0.30	0.45
Scenario 7.2 (Combination)	0.05	0.10	0.15	0.20	0.30	0.45	0.55
Scenario 7.3 (Combination)	0.10	0.15	0.20	0.30	0.45	0.55	0.60

Doses within the target toxicity interval of 20%–30% are highlighted in **bold**.

To calibrate the model parameters, only a subset of dose-toxicity and combination-toxicity scenarios are implemented to make calibration more computationally feasible. The scenarios implemented in this calibration are presented in Table 2. These scenarios cover various locations of the true MTC on the dose grid.

For the calibration of the combination parameters, another grid search is implemented, where the values of the grid again represent substantially different combination-toxicity relationships as in the monotherapy calibration. As such, the mean and the standard deviation of the prior distribution on the $\alpha_{2}$ parameter are chosen from the following two sets:

- Hyper-parameters for the combination term $\mu_{2}=\{-0.45,-0.40,\ldots ,0.20\}$ , $\sigma_{2}=\{0.1,\;0.2,\ldots ,0.7\}$ .

The parameter values selected are those that yielded the highest geometric mean of the proportion of correct MTD and MTC selection in the target toxicity interval, computed across all six of the considered scenarios which were $\mu_{2}=0.1$ and $\sigma_{2}=0.3$ .

A similar discussion on how to calibrate the model parameters for the comparative two-parameter BLRM and POCRM is provided in the supplementary materials. Note, that the model parameters for all comparator models are calibrated to optimise the combined proportion of MTD and MTC selection.

## Assessing the dose-escalation model performance

To assess the operating characteristics of the calibrated models and their expected performance, a simulation study is conducted with all three models compared. The performance of the models are measured through the following operating characteristics:

- Proportion of the dose selections in the target interval (having the DLT risk of 20%–30%);
- Proportion of the over-toxic dose selections (having the DLT risk of >30% );
- Proportion of trials that were terminated early for safety concerns.
- Proportion of patients assigned to an over-toxic dose.

A total of 1000 simulation runs were conducted for each model under each considered scenario. Note that the standard error of the Monte Carlo for this simulation study is approximated to be 0.008.

### Monotherapy and combination simulation study

The results of the simulation study investigating the monotherapy and combination therapy using the calibrated parameter values are presented in Table 3.

**Table 3.** Results of simulation study..

Scenario	1.1	1.2	1.3	2.1	2.2	2.3	3.1	3.2	3.3	4.1	4.2	4.3	5.1	5.2	5.3	6.1	6.2	6.3	7.1	7.2	7.3	Mean
Proposed Design: Three-Parameter BLRM																						
Monotherapy Part																						
Select 20%–30%	40	39	38	75	81	79	80	87	84	81	84	75	78	77	67	68	65	61	75	67	55	67
Select >30%	18	14	10	16	7	5	14	6	3	12	6	3	10	4	3	15	11	4	-	-	-	8
Early Term	42	48	52	9	12	16	2	2	5	0	1	1	0	0	0	0	0	0	0	0	0	-
Over-Toxic	7	6	6	4	3	3	3	2	1	2	1	0	1	1	0	1	0	0	-	-	-	-
Combination Therapy Part																						
Select 20%–30%	-	-	-	22	-	-	70	41	-	71	78	44	61	77	68	54	70	69	45	67	72	58
Select >30%	2	1	1	2	7	2	2	9	23	2	10	24	2	9	26	2	10	25	6	9	20	5
Early Term	99	100	100	75	93	98	28	50	77	6	12	31	1	2	6	0	0	1	0	0	0	-
Over-Toxic	4	4	3	2	6	5	1	3	8	0	1	3	0	0	2	0	0	1	0	0	0	-
Two-Parameter BLRM																						
Monotherapy Part																						
Select 20%–30%	36	37	34	72	70	69	76	74	75	74	76	75	72	73	71	59	59	62	71	70	70	64
Select >30%	18	18	16	15	19	20	16	17	16	12	13	14	13	11	12	20	21	18	-	-	-	16
Early Term	46	45	50	13	11	11	3	3	3	1	0	0	0	0	0	0	0	0	0	0	0	-
Over-Toxic	8	8	8	5	5	5	3	4	3	2	2	2	1	1	1	1	1	1	-	-	-	-
Combination Therapy Part																						
Select 20%–30%	-	-	-	43	-	-	68	39	-	76	60	35	80	72	58	64	76	66	33	66	75	58
Select >30%	24	11	7	26	41	20	25	40	44	12	35	45	3	19	37	1	6	25	1	1	8	12
Early Term	76	90	93	31	59	80	7	21	57	3	5	20	1	2	4	0	0	1	0	0	0	-
Over-Toxic	5	5	4	3	8	7	1	4	9	0	1	4	0	0	2	2	0	0	0	0	0	-
POCRM																						
Monotherapy Part																						
Select 20%–30%	86	85	87	84	84	88	77	74	70	75	73	60	75	65	57	70	62	51	71	65	63	72
Select >30%	12	14	10	16	15	12	15	14	14	11	13	18	9	11	18	9	12	16	-	-	-	13
Early Term	2	3	3	0	0	0	0	0	0	0	0	0	0	0	0	0	0	0	0	0	0	-
Over-Toxic	6	5	5	4	4	4	2	2	3	2	2	2	1	1	1	0	0	1	-	-	-	-
Combination Therapy Part																						
Select 20%–30%	-	-	-	65	-	-	85	58	-	72	73	57	69	64	65	67	64	45	62	62	49	63
Select >30%	48	24	18	12	50	27	11	28	61	8	25	37	6	20	34	5	15	29	1	14	24	18
Early Term	52	76	82	23	50	73	4	14	39	1	2	6	0	0	1	0	0	0	0	0	0	-
Over-Toxic	8	7	6	2	9	8	1	2	10	0	1	3	0	0	2	0	0	1	0	0	0	-

Columns are scenarios. Values in the table for first three rows of each block show percentage of scenarios achieving target (20%–30%) and over-toxic (>30%) selection, or early terminations (Early Term). Final row shows the proportion of patients on an over-toxic dose (Over-toxic). Mean corresponds to the geometric mean across all scenarios (excluding scenario 7 for the over-toxic selection). Results are based on 1000 simulations for each of the three approaches.

First, looking at the monotherapy part of the study, the geometric mean of the percentage of trials selecting an MTD in the 20%–30% target interval across all scenarios is the greatest in the POCRM design at 72%, followed by the proposed three-parameter BLRM at 67%, with the lowest value observed under the two-parameter BLRM at 64%. However, the proposed design reduces the percentage of over-toxic dose selections, with an average of 8% of trials selecting a monotherapy dose above the targeted 30% level. The two-parameter BLRM doubles this value at 16%, with an average of 13% of trials under the POCRM selecting an over-toxic dose. However, when the true MTD is either dose level 1 or 2, there is a trade-off between safety considerations and correctly selecting the true MTD. The proposed design terminates the trial early between 9% and 42% of trials with 38%–81% trials selecting the correct dose in the target toxicity interval and 5%–18% of trials selecting an over-toxic dose. Whereas the POCRM terminates the trial early under scenario 1 only, in which <5% of simulation runs terminated early and had an increase over the proposed design of up to 49% in terms of correct MTD selection with similar over-toxic dose selections. In contrast, under scenarios 3.1–7.3, when the true MTD is at the higher dose levels, both the proposed design and POCRM have similar early-termination proportions, with the proposed design giving consistently higher percentage of dose selections in the target toxicity interval for scenarios 3.1–5.3.

A similar comparison is drawn from the combination therapy part of the study where the two BLRM approaches have an identical average correct selection of an MTC in the target toxicity interval of around 58%, with the POCRM approach having a 63% average correct selection probability. However, both the two-parameter BLRM and POCRM have an increase in the average over-toxic selection at 12% and 18%, respectively, compared to the three-parameter BLRM at 5%. The proposed design has lower over-toxic selection across most combination-toxicity scenarios compare to the comparator models, with the two-parameter and POCRM demonstrating up to a 46% increase compared to the proposed design. The POCRM has a maximum over-toxic selection of 61%, meaning that under that scenario, over half of the simulation runs selected an unsafe dose. The proposed three-parameter BLRM has a maximum value of 26%. All three models have the same average number of patients assigned to an over-toxic dose.

## Practical implementation

In practice, a review of safety data will be conducted at a dose review meeting prior to opening any new dose level, or opening enrolment to a new part of the trial. The BLRM model will be updated using all available data and a report summarising the model output will be sent to the safety review committee prior to the meeting. These dose review meetings will be held to review if the dose level can be deemed tolerated by the participants, assess any emerging data from previous cohorts and review the updated BLRM model to set dose levels for subsequent participants in Part A and Part B. In addition, these review meetings may open enrolment to Part B if this is not yet open and declare the RP2D to open Phase IIa, the dose expansion part. These meetings will be scheduled regularly (if required) during Phase I Part A and Part B (additional meetings may take place if urgent review is required due to emerging safety data) to update the Bayesian safety model and review dosing if a participant has become evaluable for DLT assessment and has data available since the last dose review. The safety data will be assessed along with the available PK data and any additional appropriate trial data, providing quantitative support to the dose decision meetings.

## Discussion

In this work, we present an adaptation to a BLRM to incorporate the dose escalation of a monotherapy in parallel with the dose escalation of the treatment in combination with a backbone agent.

The proposed three-parameter model is beneficial over the two-parameter BLRM due to its ability to test the monotherapy and combination side by side. By conducting dose escalation of the two settings in parallel, the duration of the trial can be substantially reduced. Like the three-parameter BLRM, the POCRM also allows parallel testing of both treatments; however, results in far more selections of an over-toxic dose with similar selections of doses in the target toxicity interval. In contrast, the two-parameter BLRM defined in this work requires the completion of the monotherapy dose escalation before commencement of the testing of the combination. In this approach, the observed DLTs in the monotherapy are not utilised in the dose-escalation model for combination despite the clear link between the toxicities at monotherapy and combination. The monotherapy for the two-parameter BLRM simply reduces the dose-combination space, which although intuitive in terms of dose-toxicity monotonicity, fails to utilise the informative accrued data from the first part of the study. The two-parameter model has similar selections of the true MTD and MTC to that of the three-parameter model but, like the POCRM, increases the number of doses selected that were over-toxic.

One potential concern regarding the proposed model is the starting dose of the first cohort on combination treatment. The model, as defined, allows the first cohort of combination patients to be allocated the same dose as the next cohort for monotherapy despite the monotonicity assumption that assumes the combination is more toxic. Alongside the overdose constraint ( $c_{\mathrm{overdose}})$ an additional constraint was applied that only allowed a combination dose to be assigned if at least three DLT-evaluated patients had been assigned to the dose or higher at monotherapy. This with the fact that combination dose escalation commences from the second cohort onwards and that $\alpha_{2}$ is constrained to being positive means that in our setting the combination dose always starts at the starting dose or lower, thus the safety concerns outlined above are not an issue.

In addition, the three-parameter BLRM excludes the interaction term between the monotherapy and the combination treatment. This was motivated by Mozgunov et al.,^2^ where they demonstrate that including an interaction effect does not improve the accuracy of correct dose selection and decreases the number of patients assigned to the true MTD/MTC.

The simulation study presented focuses on a single trial setting with a parallel shift in toxicity relationship from monotherapy to combination therapy, with a 2:1 allocation ratio between monotherapy and combination therapy. In the supplementary materials, we also consider a setting with 1:1 allocation between the two parts of the trial and found very similar operating characteristics across all three approaches compared to 2:1 allocation. The proposed design was less sensitive to the allocation ratio as the combination part contributes to the model parameters for monotherapy and thus limits the impact of reducing the sample size in the monotherapy when the sample size in the combination part increases. In addition, further scenarios were explored (and presented in the supplementary material) where the combination-toxicity relationship was altered such that there was not a parallel shift in toxicity compared to the monotherapy. The proposed design proved less sensitive to the shape of the combination-toxicity in the considered scenarios, with the lowest proportion of over-toxic selections.

## Supplemental Material

sj-pdf-1-ctj-10.1177_17407745251350604 – Supplemental material for Seamless monotherapy-combination phase I dose-escalation model-based design Supplemental material, sj-pdf-1-ctj-10.1177_17407745251350604 for Seamless monotherapy-combination phase I dose-escalation model-based design by Libby Daniells, Thomas Jaki, Alimu Dayimu, Nikos Demiris, Basu Bristi, Stefan Symeonides and Pavel Mozgunov in Clinical Trials
