# Supplementary material for: Seamless monotherapy-combination phase I dose-escalation model-based design
Source: Clin Trials. 2025 Jul 12;22(4):430–41. doi: 10.1177/17407745251350604 (PMC12318161; doi:10.1177/17407745251350604)
Supplement: sj-pdf-1-ctj-10.1177_17407745251350604 – Supplemental material for Seamless monotherapy-combination phase I dose-escalation model-based design [file sj-pdf-1-ctj-10.1177_17407745251350604.pdf]

Supplementary Materials: Seamless Monotherapy-Combination Phase I Dose-Escalation Model-Based Design

1 Comparative Method: 2-Parameter Bayesian Logistic Regression Model

In order to assess the benefits of adding an additional covariate term, a comparative 2-parameter BLRM model is considered in the main text alongside the proposed 3-parameter BLRM. As proposed by Neuenschwander et al. [2008], the 2-parameter BLRM for a single agent has the following form:

$$\text{logit}(p(d_j)) = \alpha_0 + \alpha_1 \times \log(d_j/D_*). \tag{1}$$

This model is equivalent to the proposed model when $\mathbb{I}[f] = 0$, i.e. when the treatment is administered as a monotherapy. However, without the additional covariate term, this model cannot consider the combination therapy in parallel, thus the dose-escalation procedures under the 2-parameter and 3-parameter models differ. Under this comparative model, the monotherapy and combination therapy trials occur sequentially with monotherapy dose-escalation conducted, followed by the combination dose-escalation. As such, two separate BLRMs are applied: one for each part. Thus two models are defined as:

$$\text{logit}(p(d_j, \text{monotherapy})) = \alpha_0 + \alpha_1 \times \log(d_j/D_*), \tag{2}$$

$$\text{logit}(p(d_j, \text{combination})) = \alpha_2 + \alpha_3 \times \log(d_j/D_*), \tag{3}$$

where $(\alpha_0, \log(\alpha_1)) \sim \mathcal{N}(\boldsymbol{\mu_1}, \Sigma_1)$ and $(\alpha_2, \log(\alpha_3)) \sim \mathcal{N}(\boldsymbol{\mu_2}, \Sigma_2)$, with $\boldsymbol{\mu_1} = (\mu_0, \mu_1)^T$ and $\boldsymbol{\mu_2} = (\mu_2, \mu_3)^T$ and

$$\Sigma_1 = \begin{bmatrix} \sigma_0^2 & 0 \\ 0 & \sigma_1^2 \end{bmatrix}, \qquad \Sigma_2 = \begin{bmatrix} \sigma_2^2 & 0 \\ 0 & \sigma_3^2 \end{bmatrix}.$$

The monotherapy informs the combination part of the trial in only the considered dose set and does not influence the parameters α_2 and α_3 in the BLRM for the combination part. For the combination therapy, the doses considered are capped at the MTD from the monotherapy dose-escalation. Any dose higher than the MTD are not included in the dose-escalation for the combination part and are deemed in-admissible. This is to reflect the assumption that the combination toxicity cannot have a lower risk of toxicity compared to the same dose at monotherapy. Should no MTD be found under monotherapy, with the trial terminated early for safety concerns, then the trial does not continue to the combination part. Figure 1 outlines the dose-escalation procedure for monotherapy and the combination under this 2-parameter BLRM with the dose-escalation constraints outlined in the main text implemented.

2 Comparative Method: Partial Ordering Continual Reassessment Method

An alternative competing approach considered in this paper is the partial ordering continual reassessment method (POCRM) as proposed by Wages et al. [2011]. This method takes into account the unknown orderings of toxicity probabilities between doses when administered in monotherapy and when administered in combination with a backbone agent. An assumption in the proposed 3-parameter BLRM and the 2-parameter BLRM is made such that the risk of toxicity increase monotonically with the dose-level within the monotherapy setting. The same monotonicity applies within the combination setting, but also a dose at combination is assumed to be more toxic than the same dose applied as a monotherapy. However, the dose-toxicity relationship between a higher dose at monotherapy and a lower dose at combination is unknown. The POCRM is a suitable approach to handle these unknown toxicity relationships.

The POCRM approach takes the known partial dose-toxicity orderings and constructs all possible full orderings. However, when considering trial set-ups with a large number of dose-levels, the number of possible orderings can be very large. Wages and Conaway [2013] proposed an approach to select a subset of possible orderings to reduce the dimension of the problem. The selection of the subset is based on a dose-toxicity matrix which is constructed to highlight all possible dose 'combinations' which captures both the monotherapy and combination parts of the study. Wages and Conaway [2013] demonstrated that selecting just 6 orderings balanced the computational feasibility of the problem and choosing an ordering that is close to the true dose-toxicity relationship. They show that it is not required that the 'correct' ordering is included in the subset of 6 orderings, but something 'close' to it will suffice. In order to select the $M = 6$ orderings to consider, Wages and Conaway [2013] suggest selecting the orderings by: going across rows, going up columns, going up diagonals, going down diagonals, alternating down-up diagonals and alternating up-down diagonals of the dose-toxicity matrix. In the combination setting with just two-levels of the backbone agent (0 if not administered, i.e monotherapy, and 1 if administered in combination), the orderings based on going up columns and down diagonals are equivalent, leaving just $M = 5$ orderings required.

Unlike in the other two dose-escalation models presented in this paper, the POCRM design implements a one-parameter power model given by

$$p(d_{mj}) = \alpha_{mj}^{c}, \tag{4}$$

where α_{mj} are skeleton probabilities that represent the prior estimates of the probability of toxicity at dose j under a dose-ordering m. These skeleton probabilities are defined such that $\alpha_{mj} = a + b \times d_{mj}$ where a and b are constants that are calibrated to optimise operating characteristics. A Normal prior is placed on the power parameter $c \sim \mathcal{N}(\mu, \sigma^2)$ and a prior probability $p(m)$ is defined for each of the $m = 1, \ldots, 5$ orderings.

For a fair comparison, the application of the POCRM for dose-escalation of monotherapy and in combination with a backbone agent differs to that proposed by Wages et al. [2011]. The adaptation to the POCRM design involves enrolling patients in parallel onto both a monotherapy dose and combination therapy, as opposed to a single cohort of patients being enrolled onto a particular dose at each stage of the trial. However, the procedure of selecting the next dose at each stage remains unchanged compared to the original POCRM design.

Two additional constraints to those of the 2-parameter and 3-parameter BLRM are imposed for dose-escalation in order to obtain an admissible set of doses:

- No patients will be recruited to a combination dose at the start of the trial. Recruitment to combination doses will only commence when at least one combination dose is deemed safe.
- At the dose-escalation stage, if all combination doses are deemed unsafe and there are no admissible doses, then enrolment to the combination therapy part will stop. If there are admissible monotherapy doses,

then dose-escalation continues for monotherapy only. If no doses are admissible for monotherapy and combination therapy, the trial is terminated early.

Given these additional dose-escalation constraints, the process of dose-escalation under the POCRM design is outlined in Figure 2. The formulae for computing the model posterior probabilities and the posterior distribution of c are provided in Section 2.1.

2.1 Posterior Probability Formulae

Denote the probability of toxicity at dose j under ordering m as $p_m(d_j)$ then the one-parameter power model is given by $p_m(d_j, c) = \alpha_{mj}^c$, where α_{mj} is the prior probability of toxicity under ordering m at dose j. Let Y be a binary random variable, where y_j denotes the total number of toxic responses observed in patients administered dose j. Denote n_j as the number of patients administered dose j and write the complete data-set as $\mathcal{D}_j = \{y_j, n_j\}$ for dose j. After j patients have been enrolled to the study, the likelihood under a given ordering m can be written as:

$$L_m(c|\mathcal{D}_j) = \prod_{l=1}^{j} p_m(d_j, c)^{y_l}\{1 - p_m(d_j, c)\}^{1-y_l},$$

thus the posterior density for c is:

$$f_m(c|\mathcal{D}_j) = \frac{L_m(c|\mathcal{D}_j)g(c)}{\int_{\mathcal{C}} L_m(c|\mathcal{D}_j)g(c)dc},$$

where $g(c)$ is the prior distribution placed on the c parameter (a normal prior has been implemented in our comparative method). This posterior is used to obtain the posterior probabilities of each of the M orderings:

$$\pi(m|\mathcal{D}_j) = \frac{p(m)\int_{\mathcal{C}} L_m(c|\mathcal{D}_j)g(c)dc}{\sum_{m=1}^{M} p(m)\int_{\mathcal{C}} L_m(c|\mathcal{D}_j)g(c)dc},$$

where $p(m)$ is the prior probability of model m being the correct ordering. To compute the posterior probability of a toxicity under order m, we obtain the expected value of c, $\hat{c}_m$ from the posterior $f_m(c|\mathcal{D}_j)$ and implement this value in $p_m(d_j, \hat{c}_m)$.

2.2 Orderings for the Motivating Trial Example

Considering a scenario with 7 doses under investigation, as in the simulation studies presented in the main text, a dose matrix can be conducted to highlight all possible dose 'combinations' which captures both the monotherapy and combination parts of the study. This dose matrix used in the simulation study is presented in Table 1. Several of the toxicity orderings in this application are known. From Table 1, it is known that toxicity increases across rows and up columns. Given that dose-level l of monotherapy is administered ($l = 1, \ldots, J$) in combination with the backbone agent m ($m = 0, 1$), the dose administered is represented by d_{ml} which carries a probability of toxicity $p(d_{ml})$. For notation sake, this probability of toxicity $p(d_{ml})$ is written as (ml).

Table 1: Dose matrix for the POCRM used in the simulation study.

		Treatment						
		20mg QD	40mg QD	80mg QD	160mg QD	320mg QD	640mg QD	480mg BID
Backbone	1	d_{11}	d_{12}	d_{13}	d_{14}	d_{15}	d_{16}	d_{17}
	0	d_{01}	d_{02}	d_{03}	d_{04}	d_{05}	d_{06}	d_{07}

From Table 1, the known orderings between the monotherapy and combination therapy doses are represented by:

1. (01), (02), (03), (04), (05), (06), (07),
2. (11), (12), (13), (14), (15), (16), (17),
3. (01), (11),

1. (02), (12),
2. (03), (13),
3. (04), (14),

1. (05), (15),
2. (06), (16),
3. (07), (17),

where toxicity increases from left to right.

Given the trial setting and dose matrix in Table 1, the subset of 5 orderings used in the simulation study are presented in Table 2. These are found by implementing the approach by Wages and Conaway [2013].

Table 2: The subset of 5 Orderings obtained by the Wages and Conaway [2013] approach used in the simulation study.

	Ordering
Across Rows	(01), (02), (03), (04), (05), (06), (07), (11), (12), (13), (14), (15), (16), (17)
Up Columns	(01), (11), (02), (12), (03), (13), (04), (14), (05), (15), (06), (16), (07), (17)
Up Diagonals	(01), (02), (11), (03), (12), (04), (13), (05), (14), (06), (15), (07), (16), (17)
Down-Up Diagonals	(01), (02), (11), (12), (03), (04), (13), (14), (05), (06), (15), (16), (07), (17)
Up-Down Diagonals	(01), (11), (02), (03), (12), (13), (04), (05), (14), (15), (06), (07), (16), (17)

3 Calibration of Comparative Methods

The procedure for the calibration of the 3-parameter BLRM prior parameters was presented in the main text. The procedure for calibrating both the 2-parameter and POCRM parameters is the same as in the proposed method.

For the 2-parameter BLRM, the α_0, α_1 and c_{overdose} parameters are equivalent to the 3-parameter BLRM in the monotherapy setting. Thus these parameters do not need re-calibrating and are set at $\mu_0 = -1.75$, $\sigma_0 = 1.6$, $\mu_1 = 0$, $\sigma_1 = 0.2$ and $c_{\text{overdose}} = 0.4$. This leaves only the α_2 and α_3 parameters for the combination part of the study that require calibration. The following parameter space was searched for the combination parameters:

1. Hyper-parameters for the intercept $\mu_2 = \{-2.25, -2.00, \ldots, -0.50\}$, $\sigma_2 = \{1.20, 1.30, \ldots, 1.80\}$;
2. Hyper-parameters for the slope $\sigma_3 = \{0.05, 0.10, \ldots, 0.25\}$,

with $\mu_3 = 0$ and $c_{\text{overdose}} = 0.4$. The combination of the above parameters that yielded the highest geometric mean of the correct selection across the six scenarios were $\mu_2 = -1.75$, $\sigma_2 = 1.6$ and $\sigma_3 = 0.05$. These parameters closely align to those obtained for the calibrated monotherapy BLRM parameters.

The POCRM is calibrated under the combination and monotherapy setting in parallel. The POCRM consists of a one-parameter power model. The parameters that require calibration are the prior parameters on c: μ and σ^2, alongside the skeleton probabilities, which are parametrised by a and b and the overdose bound c_{overdose}. The orderings implemented for the POCRM calibration are presented in Appendix 2.1. Parameters are selected from the following sets:

- Hyper-parameters for the power parameter $\mu = \{-0.50, -0.25, \ldots, 0.75\}$, $\sigma^2 = \{0.10, 0.20, \ldots, 0.70\}$;
- Hyper-parameters for the skeleton probabilities $a = \{0.08, 0.10, \ldots, 0.16\}$, $b = \{0.02, 0.03, \ldots, 0.06\}$;
- Overdose bound $c_{\text{overdose}} = \{0.2, 0.3, 0.4, 0.5\}$.

The hyper-parameters for the skeleton probabilities are restricted in order to ensure α_{mj} values are bound between 0 and 1. The combination of parameter values that yielded the highest geometric mean of the correct selection across the six scenarios were $\mu = 0.5$, $\sigma = 0.6$, $a = 0.1$, $b = 0.05$ and $c_{\text{overdose}} = 0.5$. These values resulted in the calibrated skeleton:

$$0.15, 0.20, 0.25, 0.30, 0.35, 0.40, 0.45, 0.50, 0.55, 0.60, 0.65, 0.70, 0.75, 0.80,$$

when running monotherapy and combination parts in parallel. When considering the monotherapy alone setting, this skeleton is capped at the first seven values.

4 Monotherapy Alone Simulation Results

The model performance under the combination setting were explored in the main text, we now explore the performance under monotherapy alone. This is implemented with the calibrated parameter values under the three designs. The results for all seven data scenarios are presented in Table 3.

Table 3: Percentage of target (20-30%) and over-toxic (>30%) selection, early terminations and the proportion of patients (N) on an over-toxic dose in the monotherapy simulation study setting. The mean corresponds to the geometric mean across all scenarios (excluding Scenario 7 for the over-toxic selection). Results are based on 1000 simulations and are provided for both the proposed 3-parameter BLRM and the competing 2-parameter BLRM and POCRM approaches.

		Sc 1	Sc 2	Sc 3	Sc 4	Sc 5	Sc 6	Sc 7	Mean
**Proposed Design**	Select 20-30%	34	74	81	79	77	65	73	69
	Select >30%	20	15	14	12	9	13	-	14
	Term	46	11	2	0	0	0	0	-
	Over-toxic N	12	11	8	4	2	2	-	5
**2-Parameter BLRM**	Select 20-30%	34	73	79	81	79	67	70	69
	Select >30%	21	15	14	11	8	12	-	14
	Term	45	12	2	0	0	0	0	-
	Over-toxic N	13	11	8	4	2	2	-	5
**POCRM**	Select 20-30%	81	86	65	64	63	58	87	71
	Select >30%	12	14	16	19	20	26	-	17
	Term	7	1	0	0	0	0	0	-
	Over-toxic N	8	9	6	4	4	3	-	5

Both the proposed 3-parameter BLRM and the 2-parameter BLRM are equivalent in the monotherapy alone setting as the indicator function in the proposed design equals to zero, reducing the model to 2-parameters. As such, all three performance metrics for both approaches are equal with an average of 69% of the simulated trials selecting a dose in the target toxicity interval of 20-30%. This is paired with a 14% proportion of over-toxic selections. The early termination proportions under the BLRMs decrease as the true MTD increases in dose-level. Under scenarios 1 and 2, the starting dose of 80mg QD is an over-toxic dose, which results in a 46% and 11% of trials to be terminated early for safety concerns respectively, this comes with a lower proportion of correct selections under these scenarios. Scenarios 4-7 have no trials terminated early for the BLRMs, with an increasing number of trials selecting an under-dose. Both models have an average of 5 patients are administered an over-toxic dose across the seven scenarios, whilst the POCRM has 5 patients administered an over-toxic dose.

In contrast, the POCRM terminated trials early for safety concerns in $< 10\%$ of the simulated trials across all seven scenarios. This came with a 2% increase in the average proportion of correct selections in the target toxicity interval at 71%. This increase in the average proportion of correct selections is mainly driven by scenarios 1 and 7. In scenario 1, the proportion of correct selections increases from 34% under the BLRMs to 81% under the POCRM, while also demonstrating a decrease in over-toxic selections from 21% to 12% in this scenario. This is due to the lack of early termination of the trials under the POCRM, giving the model longer to correctly identify the true MTD. However, across the seven scenarios, the POCRM demonstrated a 3% increase in the average over-toxic selections at 17%.

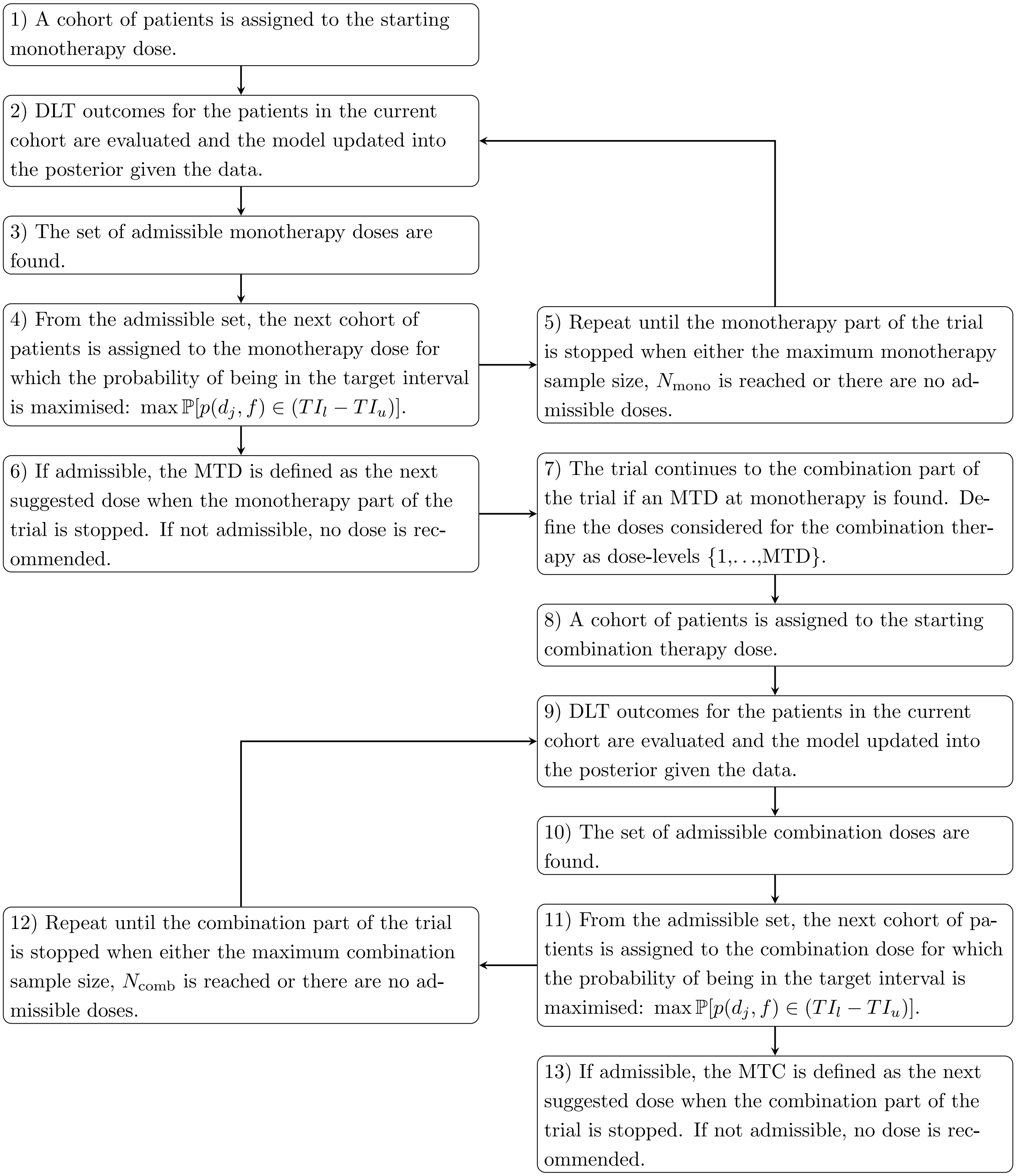

Figure 1: Dose-escalation under the 2-parameter BLRM.

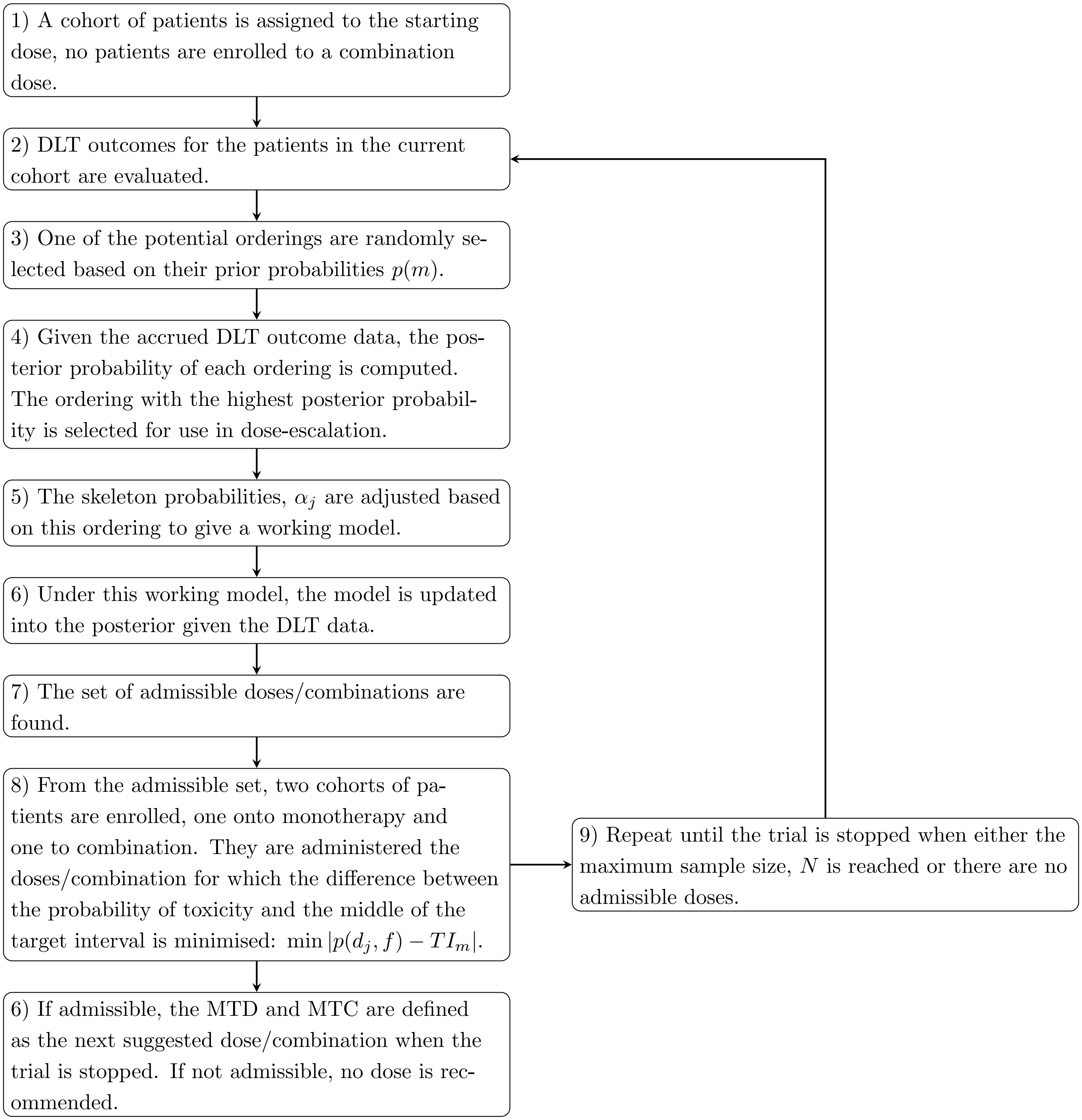

Figure 2: Dose-escalation under the partial ordering continual reassessment method.

5 Combination-Toxicity Scenarios

Table 2 in the main text details the combination-toxicity scenarios for monotherapy scenarios 4 and 7, which are implemented in the calibration of the combination parameters for the 3-parameter BLRM. Table 4 details the combination-toxicity scenarios (under all 7 monotherapy scenarios) which are implemented in the simulation study in the main text.

Table 4: Considered dose-toxicity and combination-toxicity scenarios. The target-toxicity interval is in **bold**.

Scenario	d_1	d_2	d_3	d_4	d_5	d_6	d_7
Scenario 1 (Monotherapy)	**0.30**	0.45	0.55	0.60	0.65	0.70	0.75
Scenario 1.1 (Combination)	0.45	0.55	0.60	0.65	0.70	0.75	0.80
Scenario 1.2 (Combination)	0.55	0.60	0.65	0.70	0.75	0.80	0.85
Scenario 1.3 (Combination)	0.60	0.65	0.70	0.75	0.80	0.85	0.90
Scenario 2 (Monotherapy)	**0.20**	**0.30**	0.45	0.55	0.60	0.65	0.70
Scenario 2.1 (Combination)	**0.30**	0.45	0.55	0.60	0.65	0.70	0.75
Scenario 2.2 (Combination)	0.45	0.55	0.60	0.65	0.70	0.75	0.80
Scenario 2.3 (Combination)	0.55	0.60	0.65	0.70	0.75	0.80	0.85
Scenario 3 (Monotherapy)	0.15	**0.20**	**0.30**	0.45	0.55	0.60	0.65
Scenario 3.1 (Combination)	**0.20**	**0.30**	0.45	0.55	0.60	0.65	0.70
Scenario 3.2 (Combination)	**0.30**	0.45	0.55	0.60	0.65	0.70	0.75
Scenario 3.3 (Combination)	0.45	0.55	0.60	0.65	0.70	0.75	0.80
Scenario 4 (Monotherapy)	0.10	0.15	**0.20**	**0.30**	0.45	0.55	0.60
Scenario 4.1 (Combination)	0.15	**0.20**	**0.30**	0.45	0.55	0.60	0.65
Scenario 4.2 (Combination)	**0.20**	**0.30**	0.45	0.55	0.60	0.65	0.70
Scenario 4.3 (Combination)	**0.30**	0.45	0.55	0.60	0.65	0.70	0.75
Scenario 5 (Monotherapy)	0.05	0.10	0.15	**0.20**	**0.30**	0.45	0.55
Scenario 5.1 (Combination)	0.10	0.15	**0.20**	**0.30**	0.45	0.55	0.60
Scenario 5.2 (Combination)	0.15	**0.20**	**0.30**	0.45	0.55	0.60	0.65
Scenario 5.3 (Combination)	**0.20**	**0.30**	0.45	0.55	0.60	0.65	0.70
Scenario 6 (Monotherapy)	0.02	0.05	0.10	0.15	**0.20**	**0.30**	0.45
Scenario 6.1 (Combination)	0.05	0.10	0.15	**0.20**	**0.30**	0.45	0.55
Scenario 6.2 (Combination)	0.10	0.15	**0.20**	**0.30**	0.45	0.55	0.60
Scenario 6.3 (Combination)	0.15	**0.20**	**0.30**	0.45	0.55	0.60	0.65
Scenario 7 (Monotherapy)	0.00	0.02	0.05	0.10	0.15	**0.20**	**0.30**
Scenario 7.1 (Combination)	0.02	0.05	0.10	0.15	**0.20**	**0.30**	0.45
Scenario 7.2 (Combination)	0.05	0.10	0.15	**0.20**	**0.30**	0.45	0.55
Scenario 7.3 (Combination)	0.10	0.15	**0.20**	**0.30**	0.45	0.55	0.60

6 Additional Scenarios With a Non-Parallel Shift in Toxicity Between Monotherapy and Combination Treatment

In the simulation study in the main text, combination-toxicity scenarios were constructed such that a there was a parallel shift from the toxicity at monotherapy to combination therapy. The toxicity curve was kept fixed but the doses corresponding to the toxicities were shifted along the curve based on the scenario and the location of the MTD/MTC. For example, under monotherapy scenario 4, where toxicity under dose 3 is 0.2 and 0.3 under dose 4, combination-toxicity scenario 4.1 shifts such that the toxicity at dose level 2 is the toxicity for combination dose 1, dose level 3 is the toxicity for combination dose 2, and so on. What was not considered in the main text, were scenarios where the shape of the combination-toxicity curve changed from the dose-toxicity curve at monotherapy.

We now take scenario 4 (chosen as the MTD lies in the middle two doses) and consider 4 new scenarios such that the slope of the combination-toxicity relationship changes. These scenarios are presented in Table 5. Scenario 4.4 is similar to that of 4.2, where the target-toxicity interval lies between doses 1 and 2, however, the toxicity has a higher gradient with larger doses proving more toxic than under scenario 4.2. Scenario 4.5 and 4.6 are akin to scenario 4.3 in the main text, with only the first dose in the target-toxicity interval, with all other doses over-toxic. Scenario 4.5 has dose 2 being closer to the target toxicity interval than 4.3, whilst scenario 4.6 is farther away. Under scenario 4.7, no doses are safe. The setting in which a larger monotherapy dose has no safe combination dose was not considered in the main text but could occur if the backbone agent combined with the monotherapy is highly toxic.

Table 5: Considered dose-toxicity and combination-toxicity scenarios. Doses within the target toxicity interval of 20-30% are highlighted in **bold**.

Scenario	d_1	d_2	d_3	d_4	d_5	d_6	d_7
Scenario 4 (Monotherapy)	0.10	0.15	**0.20**	**0.30**	0.45	0.55	0.60
Scenario 4.4 (Combination)	**0.20**	**0.30**	0.40	0.55	0.65	0.75	0.80
Scenario 4.5 (Combination)	**0.30**	0.35	0.45	0.55	0.65	0.75	0.80
Scenario 4.6 (Combination)	**0.30**	0.55	0.60	0.65	0.70	0.75	0.80
Scenario 4.7 (Combination)	0.40	0.45	0.55	0.65	0.70	0.75	0.80

Simulation results for the four additional scenarios are presented in Table 6. First consider scenario 4.4, we draw comparisons between the methods and to scenario 4.2 in which the MTC is located at the same dose. In scenario 4.4, dose 4 has a lower dose, closer to the target toxicity interval than scenario 4.2 which makes it more challenging to correctly select the correct MTC, thus demonstrates a 3% lower probability of correct selection for the 3-parameter BLRM and 1% decrease for both the 2-parameter BLRM and POCRM. However, the 3-parameter BLRM demonstrated lower over-toxic selections at 13% compared to 36% and 26% for the 2-parameter BLRM and POCRM respectively. The proportion of over-toxic selections have increased for all three methods compared to scenario 4.2, reflecting the difficulty in identifying the true MTC when the dose above the MTC is close but still deemed over-toxic. For the monotherapy, the 3-parameter model improves the probability of correct selection compared to the comparative models by up to 8% with a decrease of up to 7% of over-toxic selection.

Scenario 4.5 and 4.6 are akin to scenario 4.3, where only the first dose of combination treatment is safe and is the MTC. The 3-parameter BLRM increases the proportion of correct selection for both monotherapy and combination treatment compared to the 2-parameter model by up to 10%, with a substantial reduction in the proportion of over-toxic dose selections for the combination treatment of up to 17%. The POCRM has the

highest proportion of correct selections, with a 14% improvement over the 3-parameter BLRM in scenario 4.6. In scenario 4.5, dose 2 has a lower dose, closer to the target toxicity interval, thus the proportion of correct selections reduces compared to scenarios 4.2 and 4.6, with an increase in the proportion of over-toxic selections. The same holds for both POCRM and the 2-parameter BLRM, however the decrease in probability of correct selection from scenarios 4.3 to 4.5 is the smallest for the 3-parameter BLRM at a 5% reduction. In contrast, the proportion of correct selections increases under scenario 4.6 compared to scenario 4.3 as the toxicity of dose 2 is higher, making it easier for the models to distinguish the true MTC, with the 2-parameter giving marginally higher improvement between scenario 4.6 and 4.3. The proportion of over-toxic selections for the combination therapy also decrease under this scenario, with the proposed design still over a 10% reduction compared to the other approaches.

Finally, scenario 4.7 under which no combination doses are safe provides similar operating characteristics for the monotherapy as in the previous scenarios, but for combination treatment, the POCRM has a 31% increase in the proportion of over-toxic dose selections at 85%, with only 15% of trials terminating early based on the safety constraint. The 2-parameter BLRM has a lower over-toxic selection compared to the POCRM at 59%, with 41% of trials terminating early. However, the proposed design further reduces this at 54%, with 46% of trials stopping early for safety concerns.

To summarise, when the toxicity for the dose above the true MTC is closer to the target toxicity interval, the selection of the correct dose in the target toxicity interval decreases, with a greater proportion of over-toxic dose selections. The proposed 3-parameter BLRM has the smallest difference between the scenarios, thus seems less sensitive to the shape of the dose-toxicity curve. However, POCRM has higher proportions of correct selections for both scenarios 4.5 and 4.6, where only the first dose is safe. This is likely due to the lack of early termination for the POCRM compared to the BLRM approaches. However, this lack of early stopping for safety results in a higher proportion of over-toxic selections when no doses are same compared to the 2 and 3-parameter BLRMs. Overall, the proposed design continues to balance the trade-off in safety concerns and correct selections.

7 Alternative POCRM Calibration with Fixed Overdose Safety Threshold

In previous simulations, the parameters for each of the three models were calibrated in order to maximise the performance in terms of correct dose selection in order to provide a fair comparison. However, one could argue that setting a common safety threshold is sensible in order to combat safety concerns. Both the 2 and 3 parameter BLRMs have a common safety threshold of $c_{\text{overdose}} = 0.4$, whereas, previously $c_{\text{overdose}} = 0.5$ was implemented for the POCRM. We now repeat the calibration over the same grid as in Section 3 and conduct simulation studies for both monotherapy and combination therapy under the new parameters.

When c_{overdose} is fixed at 0.4, a repetition of the calibration procedure outlined in Section 3 produces the following calibrated values: $\mu = 0.25$, $\sigma = 0.1$, $a = 0.14$ and $b = 0.03$. These values resulted in the calibrated skeleton:

$$0.17,\ 0.20,\ 0.23,\ 0.26,\ 0.29,\ 0.32,\ 0.35,\ 0.38,\ 0.41,\ 0.44,\ 0.47,\ 0.50,\ 0.53,\ 0.56,$$

when running monotherapy and combination parts in parallel.

Simulation results under the monotherapy alone are provided in Table 7 and Table 8 presents the results under monotherapy and combination therapy in parallel. Under monotherapy alone, the average proportion of correct dose selection within the target toxicity interval is similar when $c_{\text{overdose}} = 0.4$ and 0.5, with only a 1% difference. However early termination decreases and over-toxic selections actually increase from 17% to 25% despite the more strict safety stopping bound. This is due to the other parameter values changing during calibration under the two safety bounds. In order to maintain the same correct selection proportion as before,

μ, σ, a and b have been altered, with the skeleton probabilities more conservative with a higher mean and lower variance on the power parameter. This led to more over-toxic selections. Similar findings are observed in the monotherapy and combination therapy simulation with lower correct selections and higher over-toxic selections.

Table 6: ercentage of target (20-30%) and over-toxic (>30%) selection, early terminations and the proportion of patients (N) on an over-toxic dose in the combination therapy setting. Results are based on 1000 simulations for each of the three approaches across the scenarios 4.4-4.7 in which there is a non-parallel shift in toxicity compared to monotherapy.

Scenario	4.4	4.5	4.6	4.7
	**Proposed Design**			
	Monotherapy Part			
Select 20-30%	81	78	74	73
Select >30%	7	4	2	2
Term	0	1	1	2
Over-toxic N	1	1	0	0
	Combination Therapy Part			
Select 20-30%	75	39	48	-
Select >30%	13	36	21	54
Term	13	25	31	46
Over-toxic N	1	4	3	10
	**2-Parameter BLRM**			
	Monotherapy Part			
Select 20-30%	76	71	74	75
Select >30%	14	15	14	13
Term	1	1	1	1
Over-toxic N	2	2	2	2
	Combination Therapy Part			
Select 20-30%	59	29	44	-
Select >30%	36	53	33	59
Term	4	17	22	41
Over-toxic N	1	4	4	10
	**POCRM**			
	Monotherapy Part			
Select 20-30%	73	64	59	62
Select >30%	12	15	19	17
Term	0	0	0	0
Over-toxic N	2	2	2	2
	Combination Therapy Part			
Select 20-30%	72	49	62	-
Select >30%	26	45	31	85
Term	1	6	7	15
Over-toxic N	1	3	2	11

Table 7: Percentage of target (20-30%) and over-toxic (>30%) selection, early terminations and the proportion of patients (N) on an over-toxic dose in the monotherapy simulation study setting. The mean corresponds to the geometric mean across all scenarios (excluding Scenario 7 for the over-toxic selection). Results are based on 1000 simulations and are provided for the POCRM approach where $c_{\text{overdose}} = 0.4$.

		Sc 1	Sc 2	Sc 3	Sc 4	Sc 5	Sc 6	Sc 7	Mean
**POCRM**	Select 20-30%	81	80	68	64	58	52	93	70
	Select >30%	19	20	21	25	33	39	-	25
	Term	0	0	0	0	0	0	0	-
	Over-toxic N	14	14	9	6	5	3	-	7

Table 8: Percentage of target (20-30%) and over-toxic (>30%) selection, early terminations and the proportion of patients (N) on an over-toxic dose in the combination therapy setting. Mean corresponds to the geometric mean across all scenarios (excluding Sc 7 for the over-toxic selection). Results are based on 1000 simulations for POCRM with fixed overdose threshold.

Scenario	1.1	1.2	1.3	2.1	2.2	2.3	3.1	3.2	3.3	4.1	4.2	4.3	5.1	5.2	5.3	6.1	6.2	6.3	7.1	7.2	7.3	Mean
Monotherapy Part																						
Select 20-30%	75	75	77	72	76	80	75	73	70	75	72	66	76	72	65	74	68	66	81	74	74	73
Select >30%	25	25	23	28	24	20	23	23	23	22	23	24	17	17	22	15	17	18	-	-	-	21
Term	0	0	0	0	0	0	0	0	0	0	0	0	0	0	0	0	0	0	0	0	0	-
Over-toxic N	10	9	9	7	6	5	4	4	4	2	2	3	1	1	1	1	1	1	0	0	0	3
Combination Therapy Part																						
Select 20-30%	-	-	-	54	-	-	77	48	-	70	62	52	70	48	55	71	54	34	67	64	43	57
Select >30%	34	16	9	22	44	24	20	39	61	18	37	44	16	35	44	10	30	42	8	18	41	25
Term	66	84	91	24	56	76	3	12	39	0	1	5	0	0	0	0	0	0	0	0	0	-
Over-toxic N	9	7	7	3	10	9	1	3	11	0	1	3	0	0	2	1	2	3	1	1	2	3

8 1:1 Allocation to Monotherapy and Combination Treatment

In the simulation study in the main text a 2:1 allocation was implemented in favour of the monotherapy. However, this implies more importance is placed on the monotherapy dose-escalation than the combination treatment. For the 3-parameter BLRM, the monotherapy informs the parameters for the combination treatment and thus having more patients on the monotherapy still contributes to the dose-escalation of the combination treatment. We now explore the setting where patients are allocated in a 1:1 ratio between the monotherapy and combination therapy. Given the same total sample size of 36 patients, the simulation study presented in this Section implements a sample size of 18 patients for both the monotherapy and combination parts of the dose-escalation study. Model parameters and scenarios implemented are identical to those implemented in the main text.

Table 9 presents the simulation results for monotherapy alone. Compared to a 2:1 allocation ratio, the probability of correct selection for the BLRMs is reduced by around 7%. This is expected due to the reduced number of patients from 24 to 18, making it more challenging to estimate the MTD. Similarly, POCRM decreases by 8%. All 3 methods give almost identical correct selection probabilities, but POCRM has 14% higher over-toxic selection, a similar finding to the 2:1 allocation simulation study, with the differences in the methods more pronounced.

Table 9: Percentage of target (20-30%) and over-toxic (>30%) selection, early terminations and the proportion of patients (N) on an over-toxic dose in the monotherapy simulation study setting. The mean corresponds to the geometric mean across all scenarios (excluding Scenario 7 for the over-toxic selection). Results are for a 1:1 allocation between monotherapy and combination treatment, based on 1000 simulations and are provided for both the proposed 3-parameter BLRM and the competing 2-parameter BLRM and POCRM approaches.

		Sc 1	Sc 2	Sc 3	Sc 4	Sc 5	Sc 6	Sc 7	Mean
**Proposed Design**	Select >30%	32	67	72	76	71	59	69	62
	Select >30%	29	22	21	16	15	21	-	20
	Term	39	11	17	0	0	0	0	-
	Over-toxic N	11	10	6	3	2	1	-	4
**2-Parameter BLRM**	Select 20-30%	38	66	73	75	72	58	72	63
	Select >30%	29	23	21	16	15	17	-	20
	Term	33	11	1	0	0	0	0	-
	Over-toxic N	12	9	5	3	2	1	-	4
**POCRM**	Select 20-30%	71	72	58	49	51	60	91	63
	Select >30%	29	28	33	42	41	33	-	34
	Term	0	0	0	0	0	0	0	-
	Over-toxic N	13	13	7	4	2	1	-	5

Table 10 presents the results for the combination and monotherapy treatment in parallel. The proposed 3-parameter BLRM has 3% decrease in correct selection at monotherapy and a 1% for combination therapy compared to 2:1 allocation in terms of correct selection. The proposed design utilises the information from combination therapy to update the monotherapy parameters, therefore, the impact of a reducing the monotherapy sample size limited. Under the 2-parameter BLRM, the impact of reducing the sample size is more pronounced with a 5% decrease in correct selection at monotherapy but the increased sample size at combination therapy results in a 5% increase in correct selections. POCRM has almost identical operating characteristics under 1:1 allocation and 2:1 allocation.

Table 10: Percentage of target (20-30%) and over-toxic (>30%) selection, early terminations and the proportion of patients (N) on an over-toxic dose in the combination therapy setting for a trial with 1:1 allocation between monotherapy and combination treatment. Standard deviations given in brackets. Mean corresponds to the geometric mean across all scenarios (excluding Sc 7 for the over-toxic selection). Results are based on 1000 simulations for each of the three approaches.

Scenario	1.1	1.2	1.3	2.1	2.2	2.3	3.1	3.2	3.3	4.1	4.2	4.3	5.1	5.2	5.3	6.1	6.2	6.3	7.1	7.2	7.3	Mean
**Proposed Design**																						
Monotherapy Part																						
Select 20-30%	40	40	36	74	80	78	77	85	85	77	80	74	76	75	62	66	60	53	64	56	44	64
Select >30%	25	19	18	20	8	7	17	6	1	13	5	1	11	4	1	14	9	2	-	-	-	7
Term	35	41	45	6	11	15	2	2	4	1	0	1	0	0	0	0	0	0	0	0	0	-
Over-toxic N	6	5	5	3	2	2	2	1	0	1	0	0	0	0	0	0	0	0	-	-	-	2
Combination Therapy Part																						
Select 20-30%	-	-	-	20	-	-	68	36	-	72	75	42	66	78	75	62	72	77	55	66	74	59
Select >30%	1	1	0	3	4	2	3	8	11	3	8	18	3	8	16	2	9	15	0	8	16	5
Term	99	99	100	77	96	98	30	56	89	8	17	41	1	3	9	0	0	1	0	0	0	-
Over-toxic N	4	4	3	3	3	3	1	3	9	1	2	5	0	1	3	0	1	2	0	0	1	2
**2-Parameter BLRM**																						
Monotherapy Part																						
Select 20-30%	34	34	33	62	62	61	66	65	66	66	64	64	68	66	64	71	69	66	63	61	64	59
Select >30%	26	27	28	28	27	29	26	26	26	21	24	23	15	16	16	5	6	8	-	-	-	19
Term	40	38	39	10	10	9	3	4	3	1	1	1	0	0	0	0	0	0	0	0	0	-
Over-toxic N	6	6	6	4	4	4	2	2	2	1	1	1	0	0	0	0	0	0	-	-	-	3
Combination Therapy Part																						
Select 20-30%	-	-	-	41	-	-	71	44	-	73	69	41	72	71	70	72	68	70	70	68	64	63
Select >30%	14	3	1	18	23	6	18	25	22	15	24	25	10	20	24	6	18	20	2	10	23	12
Term	86	97	99	41	77	94	10	31	78	2	7	34	1	2	6	0	0	2	0	0	0	-
Over-toxic N	7	6	5	4	11	8	3	6	11	1	3	6	0	2	4	0	1	2	0	0	1	4
**POCRM**																						
Monotherapy Part																						
Select 20-30%	84	85	85	86	85	87	78	75	67	76	70	62	70	67	54	65	62	54	71	60	62	71
Select >30%	14	11	12	14	14	13	12	15	16	12	15	18	12	12	19	11	11	14	-	-	-	13
Term	3	3	4	0	0	0	0	0	0	0	0	0	0	0	0	0	0	0	0	0	0	-
Over-toxic N	6	5	5	4	4	4	2	3	3	2	2	2	1	1	1	1	0	1	-	-	-	2
Combination Therapy Part																						
Select 20-30%	-	-	-	69	-	-	85	60	-	74	74	58	68	59	66	61	60	42	61	64	53	63
Select >30%	46	23	18	12	49	25	10	28	60	8	24	37	6	22	34	5	16	32	1	11	22	17
Term	54	77	82	20	51	75	5	12	40	1	2	5	0	0	1	0	0	0	0	0	0	-
Over-toxic N	8	7	6	2	9	8	1	2	10	0	1	3	0	0	2	0	0	1	0	0	0	3

9 Decision Trees

The dose-escalation behaviour of the three calibrated models is now explored through decision trees that show the recommended doses for the first three cohorts of patients, with some example escalations for a fourth cohort. This escalation is dependent on the number of observed DLTs (from zero to three) in each cohort of three patients.

9.1 Monotherapy Decision Tree

Figure 3 is the dose-escalation decision tree under the 2 and 3-parameter BLRMs and the POCRM approach under the monotherapy setting alone. From this, under the BLRMs, in the first three cohorts of patients on the trial, if no DLTs have been observed in the current cohort of patients, the decision is always to escalate to the next highest dose-level, regardless of the observed DLTs in the previous cohort. Similarly, if one DLT is observed then the model recommends administering the next cohort of patients the current dose. If two out of the three patients in the current cohort have a DLT, then the next recommended dose is often to de-escalate to the next lowest dose (or to recommend for early termination if already at the lowest dose level). The one exception to this is where no DLTs were observed in the first cohort and two in the second. In this case the model recommends treating the next cohort on the current dose. Finally, if three DLTs are observed, the recommendation is to always de-escalate or to terminate the trial for safety concerns, dependent on the observed DLTs in the first cohort. In the fourth cohort example, if the dose administered in the third cohort was the lowest dose, i.e. 20mg, and at least 2 DLTs are observed, then no dose is recommended. Similarly, if the dose in the third cohort was 40mg and 3 DLTs are observed in the fourth cohort, the trial terminates early for safety concerns.

The behaviour of the POCRM dose-escalation differs slightly to that of the BLRMs. When 0 DLTs are observed in the current cohort, in all but one case in the first three cohorts recommends escalation of the dose to the next highest level (with three DLTs observed in the first cohort being the exception). When 1 DLT is observed, the model always recommends administering the current dose to the next cohort of patients. In comparison to the BLRMs, although the same observed DLTs result in early termination of the trial in the thrid cohort when two DLTs are observed, the POCRM is far less likely to terminate early in the fourth cohort. Instead, of terminating early, the model recommends administering the lowest dose to the fourth cohort. In general, this model favours de-escalating to 20mg in most cases when 2 or 3 DLTs are observed and thus appears to be more conservative compared to the BLRMs. However, the lack of early termination, as seen in the simulation results in Table 3, results in a higher proportion of over-toxic doses selected.

9.2 Monotherapy & Combination Decision Tree

In order to conduct the dose-escalation under the monotherapy and combination treatment in parallel, fixed monotherapy data is assumed for each cohort in order to construct the decision trees. The data summary for the implemented fixed monotherapy dose-escalation is presented in Table 11. This data includes the doses administered to each cohort and the number of observed DLTs for each.

The decision trees for the POCRM and 3-parameter BLRM are constructed based on sequential dose-escalation in which the monotherapy data is fixed and dose-escalation is conducted for the combination treatment only, utilizing the fixed monotherapy data. The calibrated model parameters and study design parameters from the main text are implemented.

Under the 3-parameter BLRM, dose-escalation based on the first cohort's observed DLT outcomes is more conservative than the other approaches in terms of escalation and less conservative in terms of de-escalation. When no DLTs or one DLT is observed in the first cohort, the next cohort is administered the current dose of 80mg, whereas, under the 2-parameter BLRM and POCRM, no DLTs in the first cohort results in a dose-

Table 11: Fixed monotherapy dose-escalation data implemented in the Decision Trees for escalation of the combination data.

Cohort	Dose Administered	Observed DLTs
Cohort 1	80mg	0/3
Cohort 2	160mg	0/3
Cohort 3	320mg	1/3
Cohort 4	320mg	0/3
Cohort 5	640mg	1/3
Cohort 6	640mg	2/3
Cohort 7	320mg	1/3
Cohort 8	320mg	2/3

escalation to 160mg. For the proposed design, when two or three DLTs are observed in the first cohort, a dose of 40mg is administered to the next cohort and is thus less conservative than the other two-approaches which de-escalate to 20mg in some cases. The 3-parameter model results in early termination under only one dose-path: when three DLTs are observed in both of the first two cohorts.

For the POCRM approach, regardless of previous DLT outcomes, if no DLTs in a current cohort are observed, then the dose is escalated to the next highest level. If one DLT is observed, the current dose is administered. When two or three DLTs are observed, the model will almost always de-escalate and in some cases administered the same dose as the current cohort. The POCRM does not terminate the trial early under any DLT paths during the first three cohorts.

The dose-escalation for the combination treatment under the 2-parameter BLRM is conducted based on the MTD obtained from the fixed monotherapy data, with the dose-set implemented for escalation limited to the MTD or lower. The MTD obtained from the fixed monotherapy data was 320mg, and thus only the following set of doses were considered in the decision tree: (20mg QD, 40mg QD, 80mg QD, 160mg QD, 320mg QD). Under the 2-parameter BLRM, having observed no DLTs in the first three cohorts (under combination treatment) always results in an escalation to the next highest dose, similarly, if one DLT is observed, the next cohort receives the same dose as the current cohort. If two or three doses are observed, usually the dose is de-escalated at least one dose-level. The 2-parameter BLRM recommends early termination in three DLT outcome paths: when three out of three patients in the first cohort experienced a DLT and two or three in the second cohort also observed DLTs; and when two out of three patients in the first cohort experienced a DLT and 3 in the second cohort also observed DLTs.

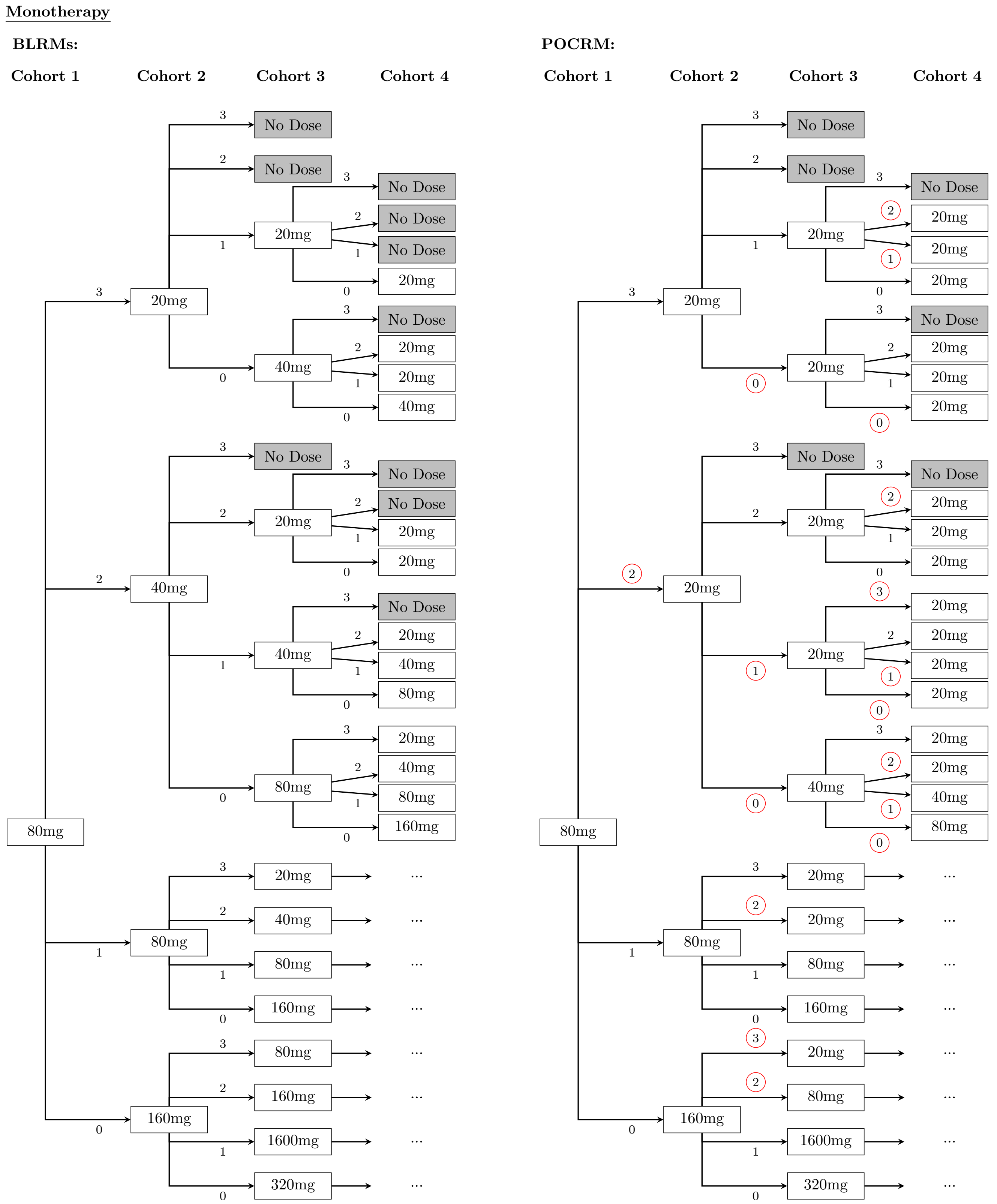

Figure 3: Decision Tree example for the first 3 cohorts of a monotherapy trial (and a selection of decisions for the 4th cohort) under the two BLRMs and the POCRM approach. Decisions are based on the number of observed DLTs (0, 1, 2 or 3 out of the cohort of 3 patients). Encircled in red are the number of observed DLTs that led to a difference between the POCRM and BLRMs dose-escalation.

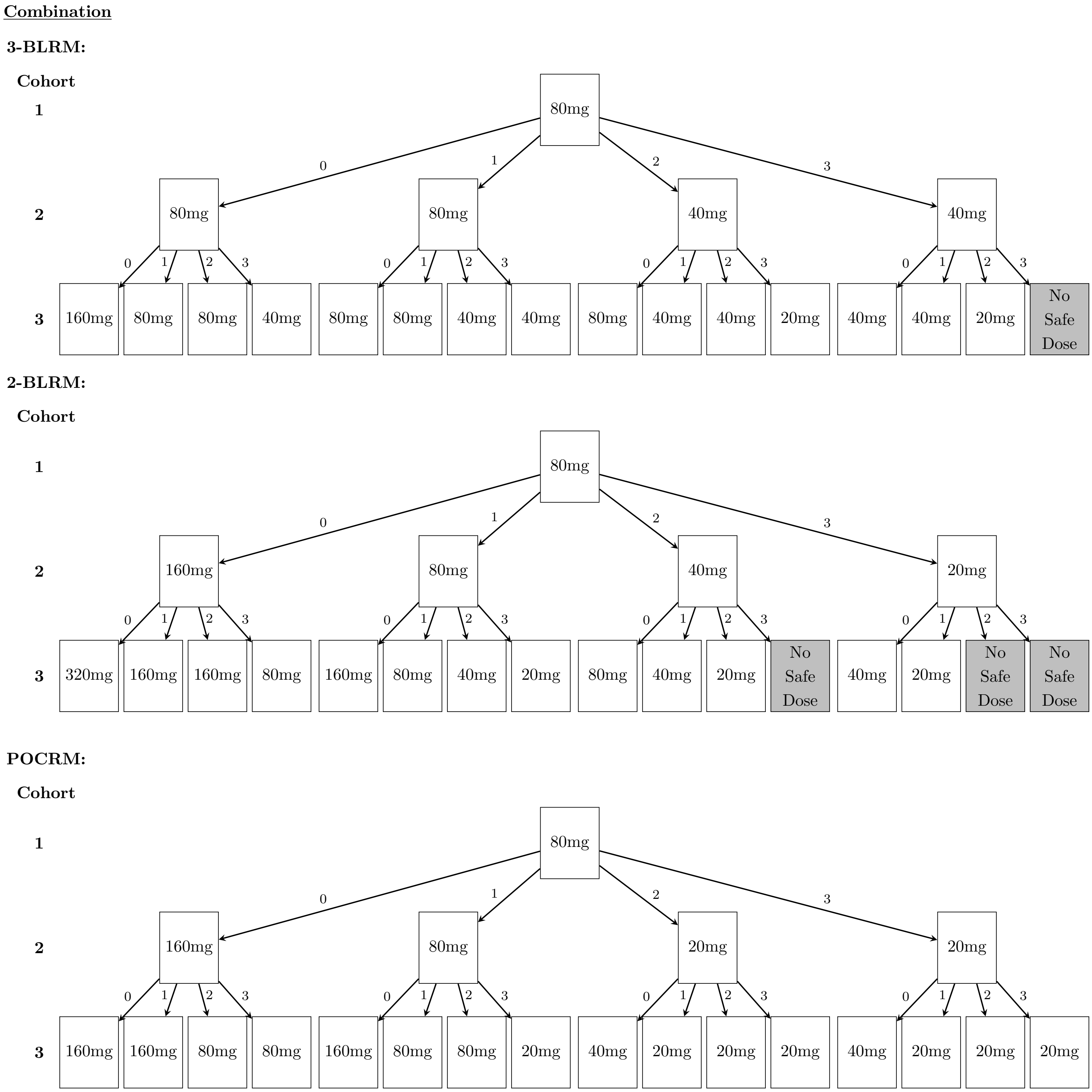

Figure 4: Decision Tree example for the first 3 cohorts of a combination trial under each of the 3 approaches. Decisions are based on the number of observed DLTs (0, 1, 2 or 3 out of the cohort of 3 patients) under fixed monotherapy data.

References

Beat Neuenschwander, Michael Branson, and Thomas Gsponer. Critical aspects of the bayesian approach to phase i cancer trials. *Statistics in Medicine*, 27(13):2420–2439, 2008. doi: https://doi.org/10.1002/sim.3230.

Nolan A. Wages, Mark R. Conaway, and John O'Quigley. Continual Reassessment Method for Partial Ordering. *Biometrics*, 67(4):1555–1563, 03 2011. ISSN 0006-341X. doi: 10.1111/j.1541-0420.2011.01560.x.

Nolan A. Wages and Mark R. Conaway. Specifications of a continual reassessment method design for phase I trials of combined drugs. *Pharmaceutical Statistics*, 12(4):217–224, 2013. doi: https://doi.org/10.1002/pst.1575.
